# Exposure of Methicillin-Resistant *Staphylococcus aureus* to Low Levels of the Antibacterial THAM-3ΦG Generates a Small Colony Drug-Resistant Phenotype

**DOI:** 10.1038/s41598-018-28283-3

**Published:** 2018-06-29

**Authors:** Alan J. Weaver, Tami R. Peters, Brian Tripet, Abigail Van Vuren, Rakesh, Richard E. Lee, Valérie Copié, Martin Teintze

**Affiliations:** 1 Department of Chemistry & Biochemistry, Montana State University, Bozeman, Montana, United States of America; 2 Department of Chemical Biology and Therapeutics, St. Jude Children’s Research Hospital, Memphis, Tennessee United States of America; 3 Present Address: Dental and Craniofacial Trauma Research and Tissue Regeneration Directorate, U.S. Army Institute of Surgical Research, JBSA Fort Sam Houston, Texas, United States of America

## Abstract

This study investigated resistance against trishexylaminomelamine trisphenylguanide (THAM-3ΦG), a novel antibacterial compound with selective microbicidal activity against *Staphylococcus aureus*. Resistance development was examined by culturing methicillin resistant *S. aureus* (MRSA) with sub-lethal doses of THAM-3ΦG. This quickly resulted in the formation of normal (WT) and small colonies (SC) of *S. aureus* exhibiting minimal inhibitory concentrations (MICs) 2× and 4× greater than the original MIC. Continuous cell passaging with increasing concentrations of THAM-3ΦG resulted in an exclusively SC phenotype with MIC >64 mg/L. Nuclear magnetic resonance (NMR)-based metabolomics and multivariate statistical analysis revealed three distinct metabolic profiles for THAM-3ΦG treated WT, untreated WT, and SC (both treated and untreated). The metabolome patterns of the SC sample groups match those reported for other small colony variants (SCV) of *S. aureus*. Supplementation of the SCV with menadione resulted in almost complete recovery of growth rate. This auxotrophism was corroborated by NMR analysis revealing the absence of menaquinone production in the SCV. In conclusion, MRSA rapidly acquires resistance to THAM-3ΦG through selection of a slow-growing menaquinone auxotroph. This study highlights the importance of evaluating and monitoring resistance to novel antibacterials during development.

## Introduction

The development of antibiotic resistance has been a concern ever since antibiotics were first discovered^1,2^. Increased usage and misuse of antibiotics over the years has resulted in increased incidence of antibiotic resistance^3,4^, triggering an urgent need to develop novel and more effective antibacterials against antibiotic resistant bacteria^5,6^. Efforts to assess how and when target bacteria are likely to develop resistance to newly developed antibacterial agents have thus become critical^7^. Representative mechanisms associated with bacterial resistance to antibiotics include chemical modification or destruction of the antibiotic, alterations to membrane permeability or efflux to prevent antibiotic accumulation within the cell, as well as modification or protection of the antibiotic’s target^8–11^. *Staphylococcus aureus*, a Gram-positive opportunistic human pathogen, has become resistant to nearly all available antibiotics, and is a major health threat^12–14^. Both hospital-acquired and community-associated *S. aureus* infections are now often caused by multi-drug resistant strains^12,15,16^. Furthermore, *S. aureus* bacterial genomes appear to contain antibiotic resistance genes even prior to exposure to novel antibacterials^17–19^, highlighting the importance of evaluating the resistance potential of any novel antibacterial prior to clinical use.

We have reported that the polyguanide compound trishexylaminomelamine trisphenylguanide (THAM-3ΦG) exhibits promising effectiveness as a novel antibacterial against *S. aureus*, including the methicillin-resistant strain USA300 (MRSA)^20^. THAM-3ΦG was initially selected from a library of guanide and biguanide-containing compounds that were screened due to structural similarity to biguanides, such as chlorhexidine and alexidine, which have been used for decades in topical applications to treat both Gram-positive and Gram-negative bacterial infections, with minimal development of antibiotic resistance in the pathogens^21^. However, concerns about bacterial resistance to these compounds are emerging^22,23^. THAM-3ΦG was found to be selectively active against *S. aureus*, including MRSA (MIC 2 mg/L). Its mechanism of action seems to be due in part to its ability to disrupt cell membranes^20^, and is similar to that of chlorhexidine^24^. In this study, we sought to investigate the potential for MRSA to develop resistance against THAM-3ΦG and to characterize the phenotype of resulting antibacterial-resistant *S. aureus* isolates.

## Results

### Sub-lethal THAM-3ΦG Dosing of MRSA Results in Elevated MIC and Altered Phenotype

**Figure 1:**
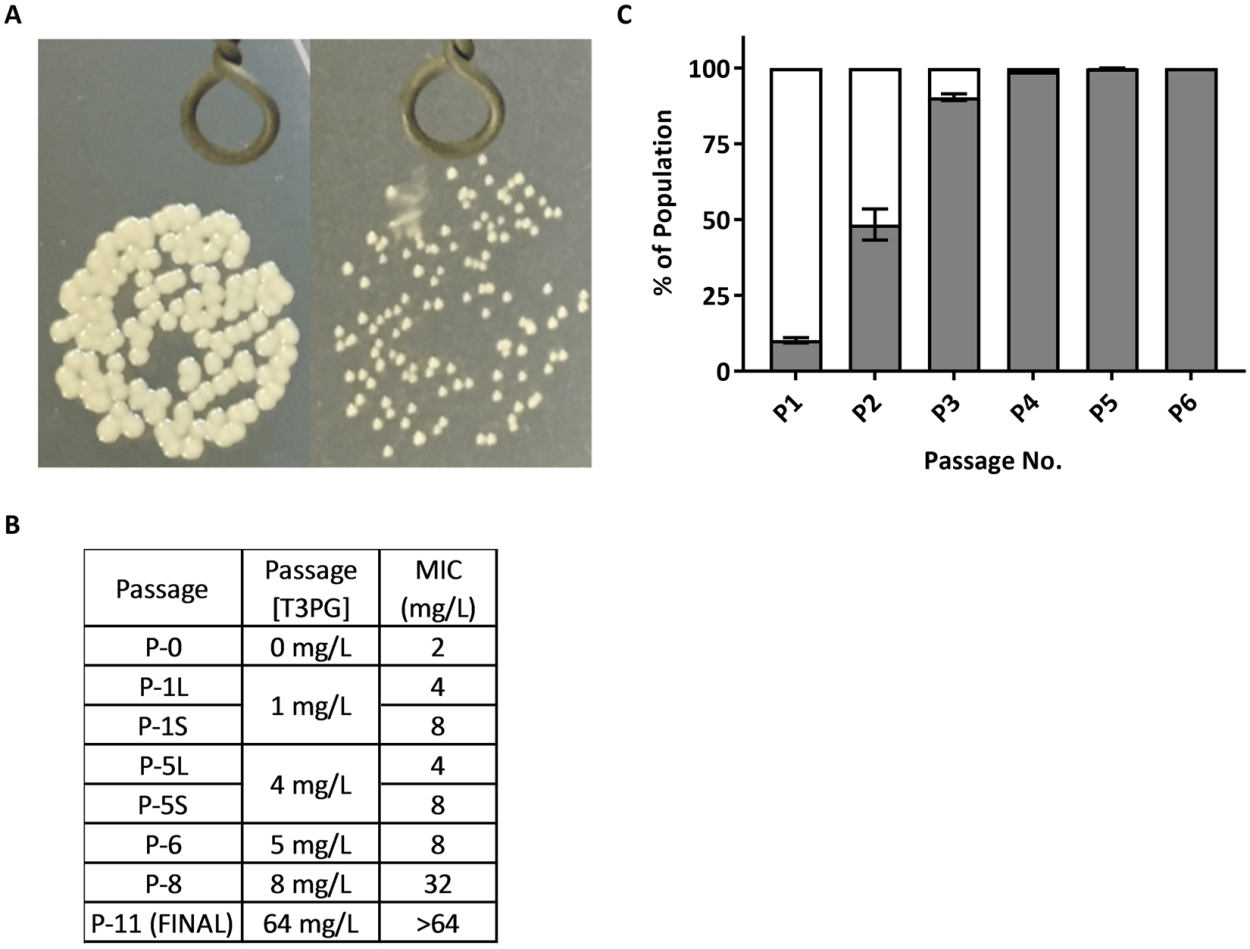
MRSA Resistance Development against THAM-3ΦG. (**A**) Left: WT colonies. Right: Small colony (SC) phenotype developed upon prolonged exposure to THAM-3ΦG. (**B**) MIC testing on individual colony isolates revealed a mixed population of large (L) and small (S) colony forming cells resistant to different concentrations of THAM-3ΦG. (**C**) Further cell passaging of this heterogeneous population as a function of increasing THAM-3ΦG resulted in a homogeneous population of small colony forming cells (shaded area) by passage 6 (P-6).

MRSA cultures were observed to develop resistance rapidly upon exposure to sub-lethal doses of THAM-3ΦG. Following growth to mid-log phase in THAM-3ΦG at 1 mg/L (0.5X MIC), a heterogeneous bacterial population containing two different cell phenotypes was observed following growth of drop-plated cell dilutions on agar: normal-sized colonies (WT) and small colonies (SC) (Fig. 1A). MIC testing was carried out on individual isolates of the WT and SC phenotypes and revealed a 2-fold increase in the MIC of the WT (P-1L; to 4 mg/L) and a 4-fold increase for the SC (P-1S; to 8 mg/L) compared to the original (P-0) WT MIC of 2 mg/L (Fig. 1B and Supplemental Table S1). Although the MICs remained constant over the next 4 cell passages with increasing doses of THAM-3ΦG, the proportion of the SC phenotype significantly increased with each cell passage and dominated the population by passage 3 (Fig. 1C). While normal-sized colonies were still visible at 4 mg/L (equivalent to the new MIC), at 5 mg/L (passage 6) 100% of the colonies exhibited the small colony morphology characteristic of the SC phenotype. This suggested that the resistance mechanism induced in the WT cells was no longer effective at providing WT cell survival at THAM-3ΦG concentrations greater than 4 mg/L.

**Figure 2:**
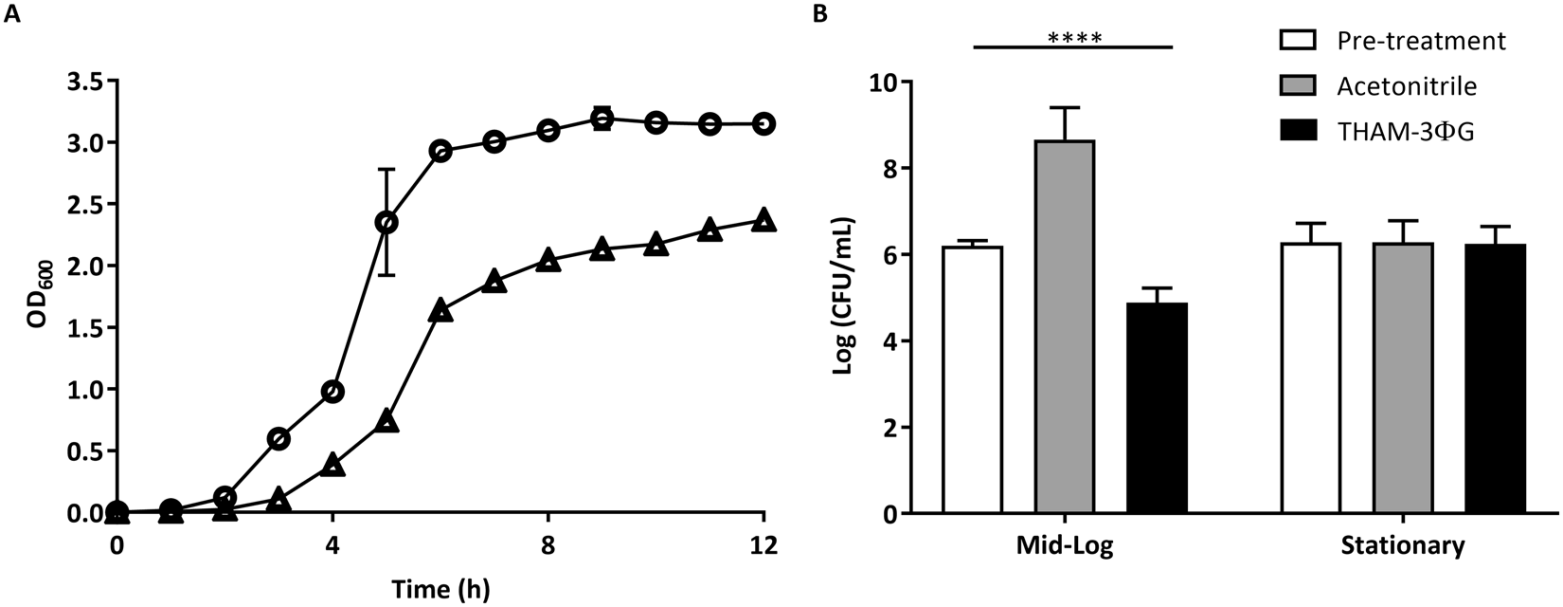
(**A**) Growth Profiles of WT and SCV Isolates. SCV (triangles) compared to the WT control (circles). A significant difference in OD_600_ between WT and SCV was observed from 3 through 12 hours (p < 0.0001). (**B**) Activity of THAM-3ΦG against Growing, but not Stationary Cells at 8 mg/L (4X MIC). CFU before incubation (white), after incubation without THAM-3ΦG (gray), and after incubation with THAM-3ΦG (black). Asterisks indicate significant changes (p < 0.0001) from pre-treatment levels. Statistical analysis are based on one-Way ANOVA.

The SC cells were passaged further with increasing doses of THAM-3ΦG until reaching the compound’s solubility limit of 64 mg/L. Unlike the WT cells, which were removed from the population following growth at THAM-3Φ concentrations above 4 mg/L, the SC cells continued to adapt to increasing doses of THAM-3ΦG, as evidenced by the increased MIC observed at passages 8 and 11 (32 and >64 mg/L, respectively; Fig. 1B). Following growth at 64 mg/L THAM-3ΦG, MICs could no longer be determined due to the solubility limit of THAM-3ΦG at 64 mg/L. The SC isolate obtained from the final passage (passage 11) was used in all subsequent studies, and is referred to as the SCV strain. The growth rate of the SCV strain was compared to that of the WT strain to assess any fitness costs associated with resistance development (Fig. 2A). The SCV strain displayed a reduced growth rate compared to WT, marked by a slightly longer lag phase and a lower cell density at the end of the exponential growth phase. Following 12 hours of incubation, SCV cultures were still notably less dense than the WT based on OD_600_. This observation correlates with the SC phenotype shown in Fig. 1A, and indicates that the small colony THAM-3ΦG resistant cells exhibited slower growth compared to the WT strain. THAM-3ΦG was effective against mid-log phase WT cells but not stationary phase WT cells (Fig. 2B), suggesting that THAM-3ΦG is only effective against actively dividing cells. Therefore, the slower growth rate of the SCV isolate would appear to be advantageous for the development of resistance against THAM-3ΦG. The SC phenotype in colonies isolated during the resistance assay at 1 mg/L, 4 mg/L and 64 mg/L THAM-3ΦG was stable for 11 passages in the absence of THAM-3ΦG, suggesting that the resistance was conferred by a mutation that occurred early in the selection process. However, additional increases in MIC at passages 8 and 11 in the resistance assay without further changes in colony size imply that a secondary resistance mechanism may have developed at higher THAM-3ΦG concentrations that was not reflected by any further decrease in growth rate.

### Metabolic Profiling of WT and SCV Isolates with and without THAM-3ΦG Treatment

**Figure 3:**
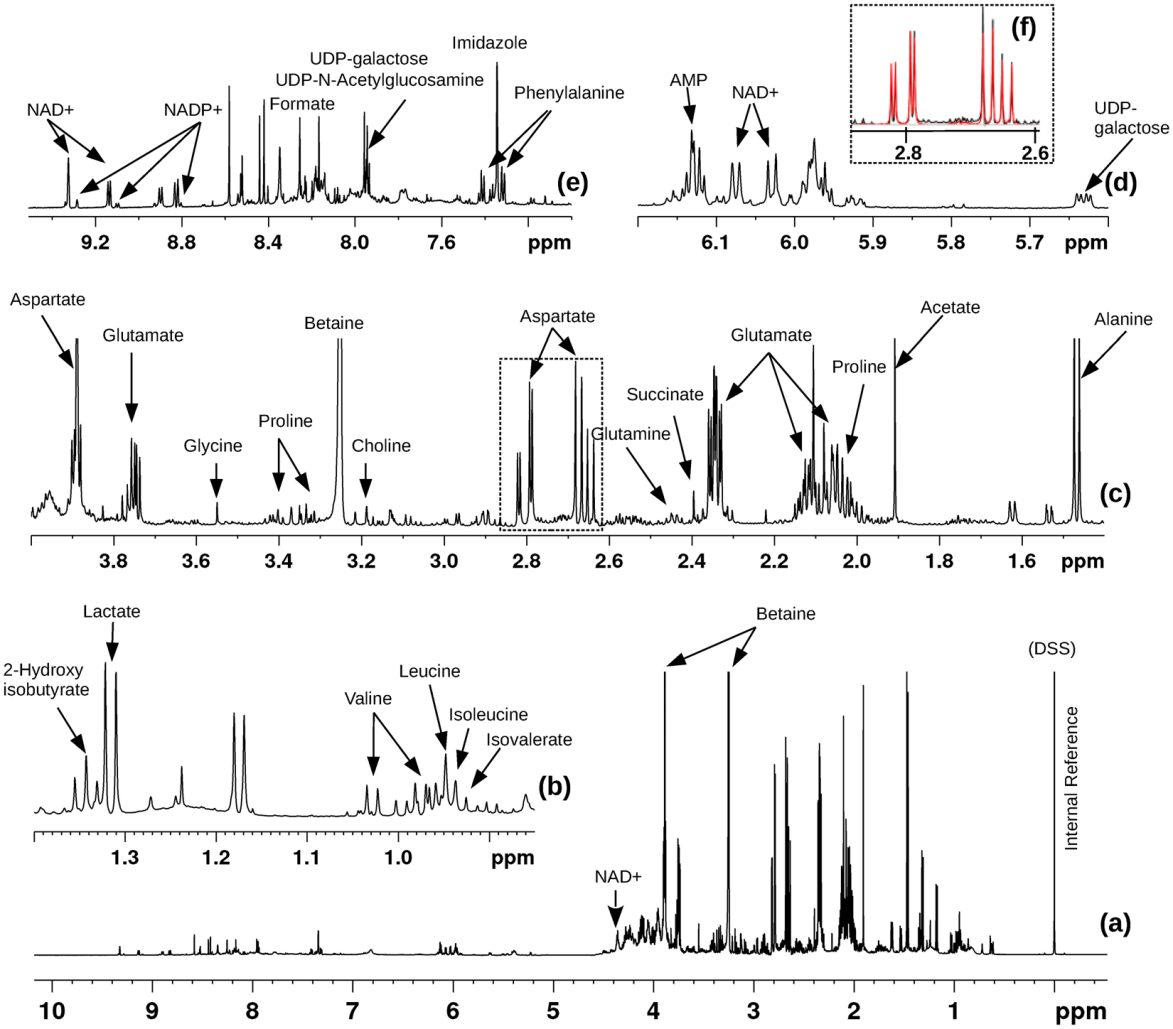
(**a**) Characteristic 1D ^1^H NMR spectrum of the protein- and lipid-free intracellular metabolite extract of wild-type *S. aureus* recorded at 600 MHz (^1^H Larmor frequency) magnetic field strength with expanded regions of the spectrum displayed in panels (**b**–**e**). The NMR signals assigned to several metabolites are indicated. The insert (panel f) displaying the spectral patterns spanning the chemical shift range 2.6 to 2.85 ppm highlights how well the spectral features of aspartate can be fitted to its characteristic spectrum annotated in the Chenomx^TM^ database (fit is in red) and compared to the experimental spectrum (shown in black). All NMR chemical shifts of the metabolite resonances are referenced to the DSS (sodium salt of 4,4-dimethyl-4-silapentane-1-sulfonic acid) signal set at 0 ppm.

To elucidate the metabolic adaptations accompanying drug resistance that evolves upon exposure to sub-lethal doses of THAM-3ΦG, the metabolic profiles of control (non-treated) and THAM-3ΦG treated WT and SCV isolate cultures were investigated using 1D ^1^H solution NMR spectroscopy. 1D ^1^H NMR spectra were recorded on intracellular metabolite sample mixtures on MSU’s 600 MHz (^1^H larmor frequency) spectrometer. Metabolites were identified and quantified by fitting the spectral patterns, chemical shift, and spectral intensity to reference spectra of small molecule metabolites using Chenomx^TM^ software^25^ and its accompanying metabolite library for 600 MHz (^1^H Larmor frequency) NMR magnetic field strength (Fig. 3). The Chenomx^TM^ metabolite profiling approach enabled identification of 30 metabolites and deconvoluted the contributions of different metabolites to potentially overlapping signals^26^.

**Figure 4:**
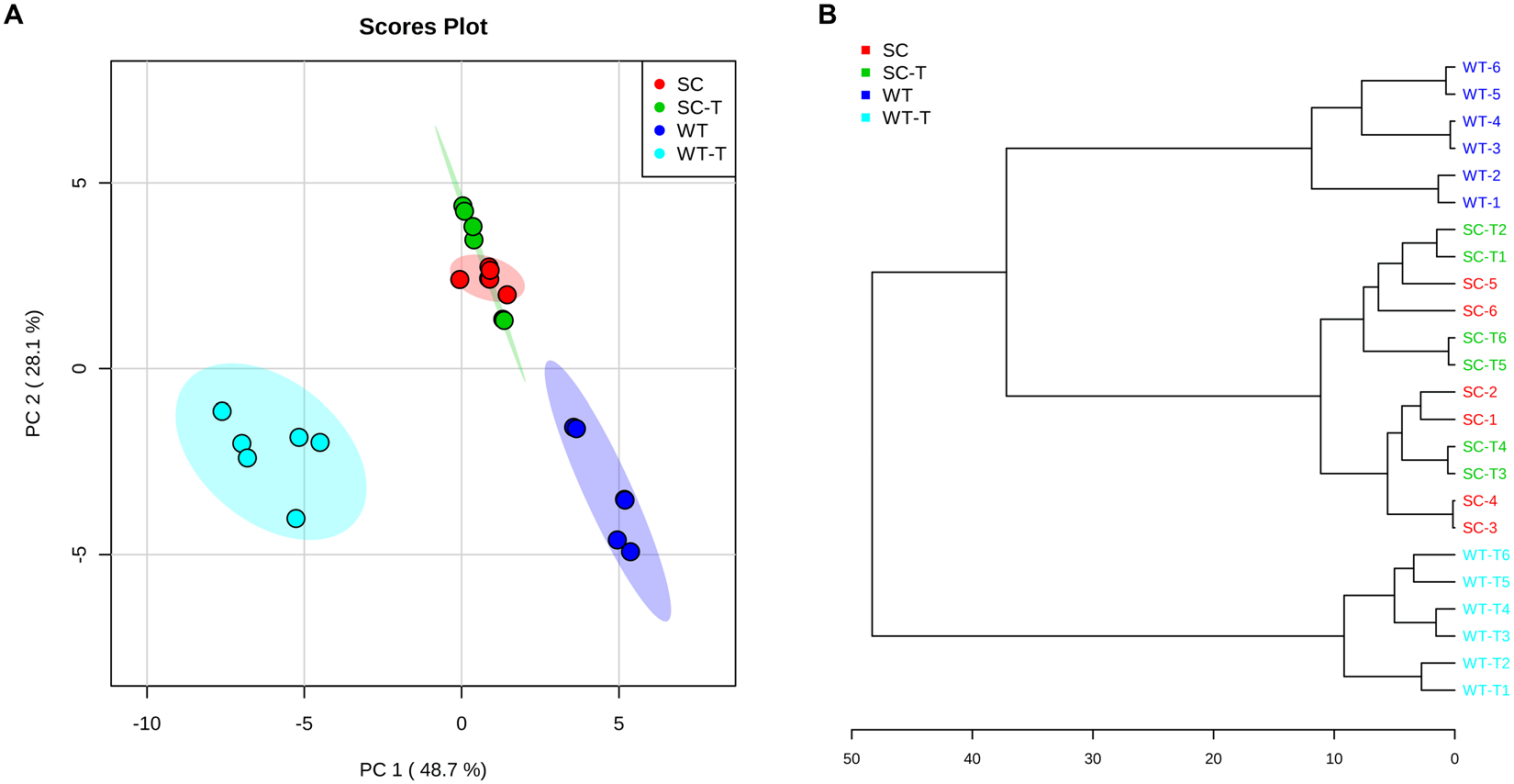
Multivariate Statistical Analysis of WT and SCV Isolates Treated and Untreated with THAM-3ΦG. Intracellular metabolites were collected for WT and SCV isolates treated (T) or untreated with 2 mg/L THAM-3ΦG for 1 hour. (**A**) 2D-PCA plot demonstrating sample separations based on distinct metabolic profiles and with clustering patterns highlighted with 95% confidence intervals as shading; PC1 and PC2 accounted for 76.8% of the variance. The 2D-PCA plot revealed three significantly distinct metabolic profiles: WT (blue), WT-T (cyan), and SC/SC-T (red/green). (**B**) Hierarchal clustering supports the separation of these groups based on Euclidean distance measurements and Ward based clustering analysis of metabolite levels. The data are representative of three biological replicates with two technical replicates of each treatment type.

From the initial profiles, three distinct cell groups were identified as being metabolically distinct in the principal component analyses (PCA), with the SCV control and treated clustering as a single group and separating from the other two groups in the principal component 2 (PC2) dimension of the 2D-PCA plots (see Fig. 4A and Supplemental Table S2 for accompanying PCA loading factors). Distinct metabolite clusters for the different *S. aureus* cell samples were further revealed via hierarchical clustering analysis, which indicated that THAM-3ΦG-treated WT cells separate from control WT and from SCV samples (Fig. 4B), as observed in the 2D-PCA plots and separation along the PC1 axis. To further understand the significance of the metabolic changes observed upon cell exposure to THAM-3Φ in terms of the antimicrobial resistance phenotype of the WT cells, additional multivariate statistical analysis was performed to identify the specific metabolite level changes accounting for the differences between control and THAM-3ΦG-treated WT cells exclusively.

### Initial Resistance Mechanisms Following THAM-3ΦG Treatment of Wild-type

**Figure 5:**
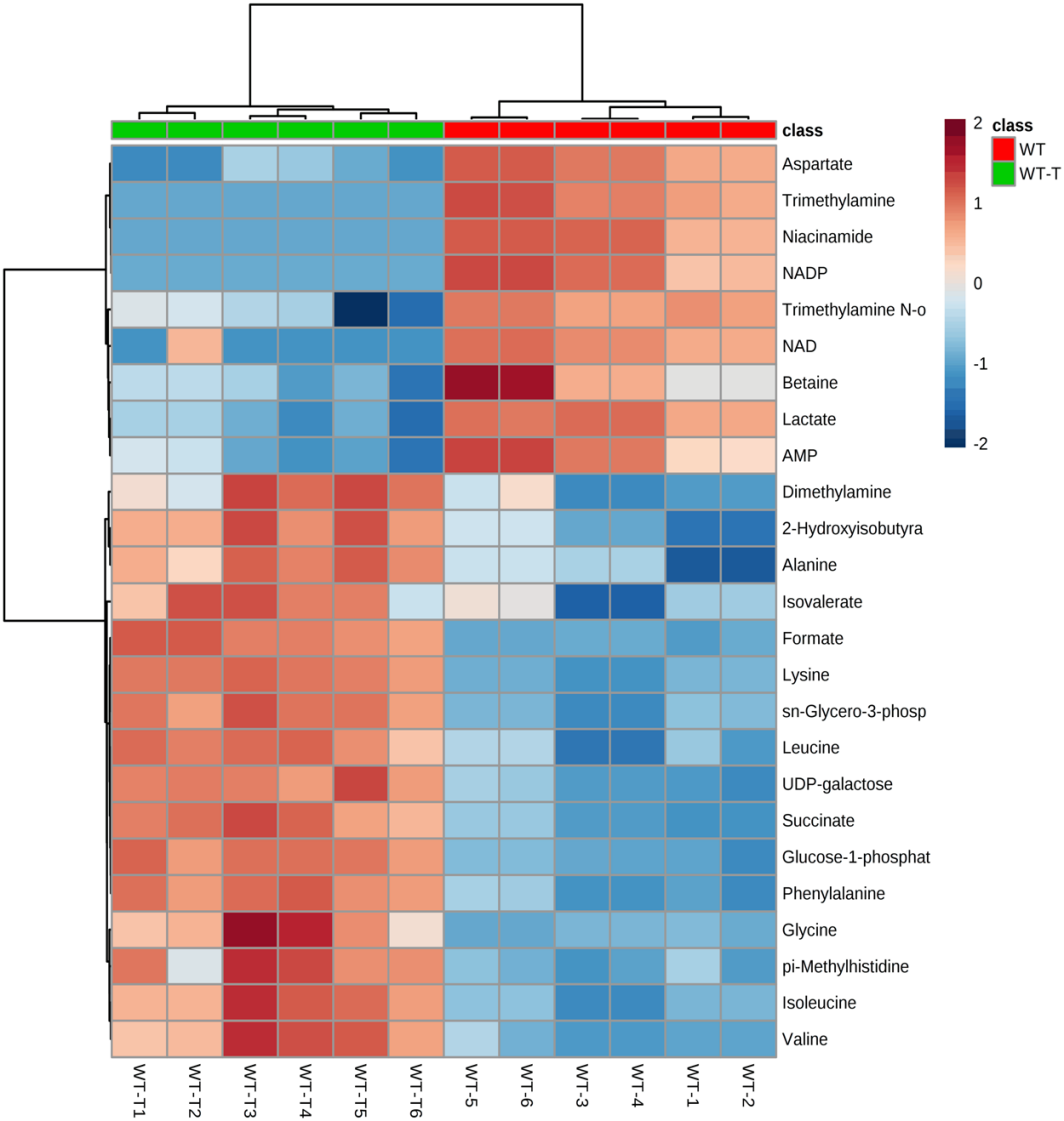
Multivariate Statistical Analysis of Control WT and THAM-3ΦG-Treated WT Metabolic Profiles. Heatmap depicting the 25 metabolites contributing most significantly to the metabolic differences between WT (red) and WT THAM-3ΦG Treated (WT-T) (green) samples (based on t-test, p < 0.01). Hierarchal clustering analysis: distances were assessed using a Euclidean correlation and clustering as established by the Ward algorithm. The data are representative of three biological replicates with two technical replicates of each treatment type.

Multivariate statistical analysis of control WT and THAM-3ΦG treated WT samples, using the hierarchal clustering and heatmap analysis module of MetaboAnalyst^27^, revealed sets of intracellular metabolites whose concentrations changed significantly following treatment with THAM-3ΦG compared to WT control (Fig. 5). Of the 25 intracellular metabolites contributing most significantly to the separation of these two groups, numerous amino acids were observed to be in relatively higher concentrations in THAM-3ΦG treated WT cells compared to control WT cells. Aspartate and glutamate were an exception and were found in relatively lower concentrations in THAM-3ΦG treated WT cells compared to WT control groups.

The stability of the SC phenotype when cultured in the absence of THAM-3ΦG selection, and the 16-fold increase in MIC achieved in the SCV after they had become the predominant phenotype suggest additional mechanism(s) of resistance developed at higher THAM-3ΦG concentrations. To further evaluate the underlying metabolic features of these mechanism(s), the metabolic profiles of SCV and WT strains grown to mid-log phase were compared using multivariate statistical analysis.

### Small Colony Variant Phenotype as the Primary Mechanism of Resistance against THAM-3ΦG

**Figure 6:**
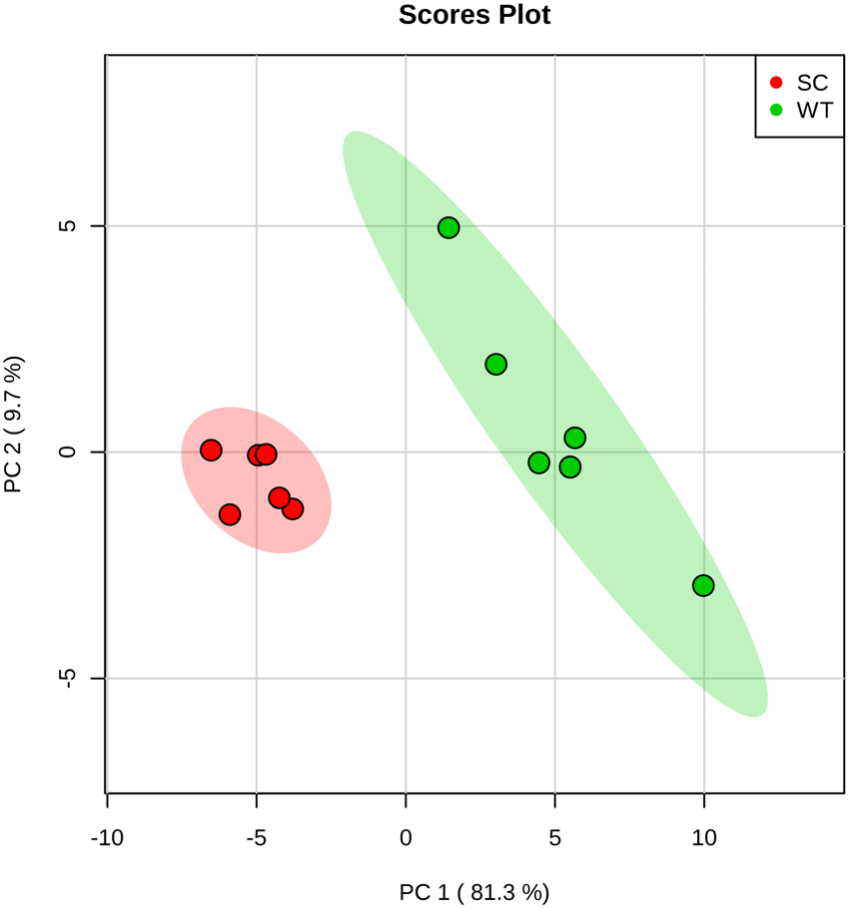
Two-Dimensional Principal Component Analysis (2D PCA) of the Intracellular Metabolic Profiles of WT and SCV Isolates Grown to Mid-Log Phase. WT (green) and SC (red), each at mid-log growth phase, exhibited distinct metabolite profiles, with principal component −1 (PC-1) accounting for 81.3% of the variance. A total of six biological replicate samples was collected for each WT and SC cell isolate.

**Figure 7:**
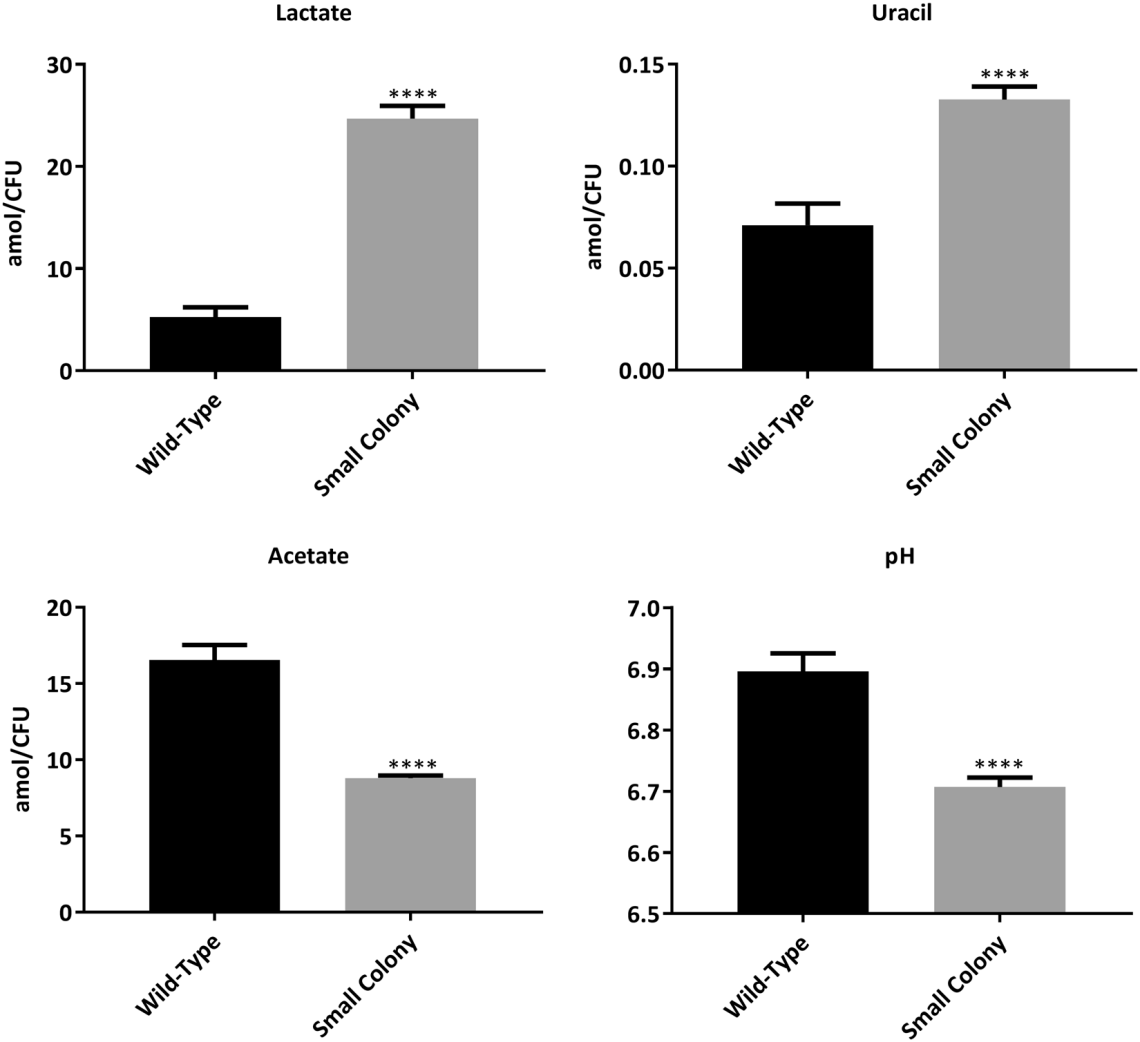
Extracellular Metabolites and pH of WT and SCV Isolates in Mid-Log Phase. Lactate and uracil concentrations were significantly higher in the extracellular environment of the SC, while acetate levels and pH were significantly lower. Significance is based on t-test (p < 0.0001). Values were collected from a minimum of 5 biological replicates.

Due to the differences in growth rate between the WT and SCV samples, isolates were cultured to the mid-log phase of each strain and both intracellular and extracellular metabolites were extracted and analyzed by NMR. 2D-PCA plots of resulting intracellular metabolite profiles separated WT from SCV along the principal component 1 (PC1) axis (Fig. 6 and Supplemental Table S3 for PCA loading factors). A general increase in the relative abundance of amino acids and many other metabolites was seen in WT THAM-3ΦG treated samples (Fig. 5), with aspartate (Asp) and glutamate (Glu) being notable exceptions. Polar extracellular metabolite profiles were also analyzed using 1D ^1^H NMR. Spectral analysis of the extracellular small molecule mixtures revealed a select number of NMR resonances which significantly differed in signal intensity between the WT and SCV groups. These were subsequently assigned to specific metabolites and quantified using Chenomx^TM^. Interestingly, high levels of extracellular lactate were detected in the SCV cells grown to mid-log phase (Fig. 6), a feature which is not expected to occur until later growth stages where the bacterial cells begin to undergo anaerobic metabolism following reduced oxygen availability at increased cell density^28,29^. Acetate was found in significantly lower concentrations in the SCV media compared to WT (Fig. 7), suggesting a shift to aerobic glycolysis and decreased TCA cycle activity, consistent with lower pH and increased extracellular lactate. Uracil levels were higher in both the intra- and extracellular metabolite fractions of the SCV strain (Fig. 7), which would be consistent with reports that some *S. aureus* SCV isolates are known to be thymidine-dependent due to an impaired thymidylate synthase, which is required for the conversion of uracil to thymidine^30,31^. Collectively, these findings led us to investigate whether the THAM-3ΦG resistant SCV strain was deficient in menaquinone, haemin, or thymidine biosynthesis.

### Supplementation with Menadione, but not Haemin or Thymidine, Leads to SCV Growth Recovery

**Figure 8:**
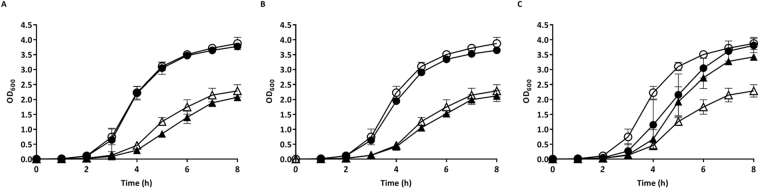
Identification of the Auxotrophism Leading to SCVs Against THAM-3ΦG. WT (○) and SCV (∆) were supplemented (●, ▲) with either (**A**) Haemin at 0.25 mg/L, (**B**) Thymidine at 100 mg/L, or (**C**) Menadione at 2 mg/L.

To further explore the nature of possible gene mutation(s) that could lead to development of an SCV phenotype following exposure to THAM-3ΦG, a series of supplementation assays were conducted based on reported SCV auxotrophs for haemin, thymidine, and menadione^31–34^. As seen in Fig. 7, supplementation with haemin (Fig. 8A) and thymidine (Fig. 8B) had no effect on the growth rate of the SCV isolate compared to WT. However, SCV supplemented with menadione showed a similar growth profile to WT supplemented with menadione (Fig. 8C), indicating that the SCV may have a reduced biosynthesis of menaquinone. Menadione is a precursor for the synthesis of menaquinone, an electron transporter that is vital to ATP production, and would therefore promote better growth of these auxotrophic bacteria^35^.

**Figure 9:**
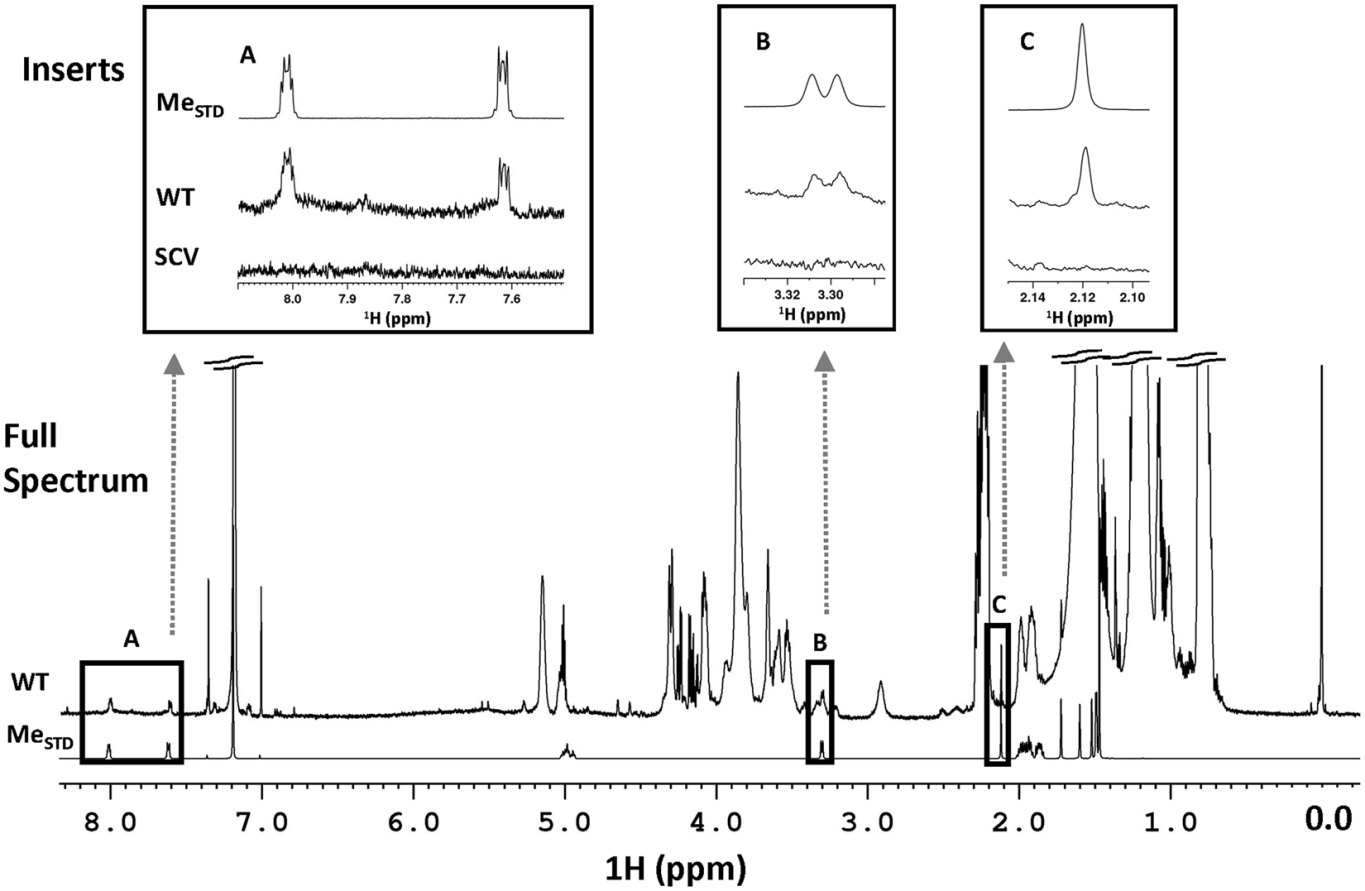
NMR Analysis of Menaquinone within WT & SCV Isolates. 1D ^1^H**-**NMR spectra of the WT and SCV non-polar metabolites were aligned with the spectrum of a menaquinone standard. Several signals in menaquinone are present in the WT spectra, but were completely absent in the SCV spectra (insets **A**–**C**). The full spectrum of SCV is not shown.

Few notable differences were observed upon comparison of the NMR spectra of the non-polar metabolite extracts from WT and SCV, apart from some NMR signals visible in the NMR spectra of WT samples being absent in the spectra of SCV samples (Fig. 9). To establish whether this was due to a lack of menaquinone production, the NMR spectra of WT and SCV isolates were compared to that of a menaquinone standard (Fig. 8). These data demonstrated that the missing NMR signals in the original SCV spectra correspond to those in the spectrum of menaquinone (Fig. 9).

### Sequencing of the *men* operons

The first six enzymes of the menaquinone biosynthesis pathway, which synthesize the double ring structure of menaquinone from chorismate are encoded in two operons, *menFDHB* and *menEC*. All six genes coding for these enzymes were sequenced from the WT and three independent clones each of SCV isolated at 4 mg/L and 64 mg/L THAM-3ΦG. No mutations in any of these genes were found compared to the WT reference genome sequence from MRSA strain USA 300. The regions with unambiguous sequence data included 160 bp upstream of the start codon of the *menEC* operon and 280 bp upstream of the *menFDHB* start codon.

## Discussion

While THAM-3ΦG displayed initial efficacy as a novel antibacterial against MRSA^20^, its potential utility appears to be compromised by the ability of MRSA to rapidly develop resistance following exposure to sub-lethal doses of THAM-3ΦG. This resistance likely occurs through at least two mechanisms. One of these is the development of an SCV phenotype with a slower growth, and this correlates with THAM-3ΦG appearing to be active only against actively dividing cells (Fig. 2). This SCV phenotype was stable during prolonged culturing without selection by THAM-3ΦG, suggesting that the resistance was conferred by a mutation that occurred early in the selection process. Additional selection with higher concentrations of THAM-3ΦG increased the MIC 16-fold, and this highly resistant SCV population was also stable in the absence of selection, suggesting a second resistance mechanism resulting from another mutation(s).

NMR metabolomics was used to investigate the underlying mechanisms of resistance. PCA and hierarchal clustering analysis of the NMR metabolic profiles revealed 3 distinct cell populations (Fig. 4), confirming that there were multiple variables contributing to the reduced susceptibility of MRSA to THAM-3ΦG. Furthermore, the lack of separation between the SCV control and SCV THAM-3ΦG-treated samples suggests that the resistance mechanism developed by the SCV is constitutively expressed, rather than being upregulated only upon exposure to THAM-3ΦG. Additional multivariate statistical analysis was performed to identify the specific metabolite level changes accounting for the differences between control and THAM-3ΦG-treated WT cells. Heat map analysis of intracellular metabolites revealed that the metabolic differences between the WT and WT-T cells was mostly due to fluctuations in amino acid levels, which were all elevated in the WT-T except for aspartate (Asp) and glutamate (Glu). Decreased levels of amino acid catabolism observed in SCV phenotypes have been attributed to decreased utilization of the TCA cycle in conjunction with anaerobic modes of growth^34^. This observation is also consistent with observations in a previous study of *S. aureus* treatment with daptomycin, another cationic antibacterial compound whose mechanism of action is membrane disruption^36^. The structure and physical properties of THAM-3ΦG suggest that it may work through a similar mechanism, partitioning into the lipid bilayers that make up *S. aureus* cell membranes and causing membrane leakage, as observed previously^20^. Following THAM-3ΦG treatment, the cells may not be able to catabolize amino acids (and potentially other carbon sources) for energy production as efficiently as untreated WT cells. Asp and Glu may be the exceptions, because they require only single-step modifications (transaminase or dehydrogenase reactions) for further catabolism via entry into the tricarboxylic acid (TCA) cycle; this might allow cells to use Asp and Glu for energy (ATP) production more efficiently compared to the catabolism of other amino acids^37,38^. Interestingly, Asp may also be consumed for the synthesis of lysine (Lys) as part of a *S. aureus* THAM-3ΦG resistance mechanism; this would be consistent with observations that *S. aureus* can upregulate the production of lysyl-phosphatidylglycerol (L-PG) upon exposure to cationic antimicrobials^39–41^. L-PG is a positively charged lipid, and its increased production relative to PG and other negatively charged lipids would effectively reduce the negative charge of the bacterial cell membrane, thereby reducing the membrane binding affinity for cationic antimicrobial compounds like THAM-3ΦG^42,43^. This L-PG mediated resistance mechanism has been observed in *S. aureus* strains that develop resistance to cationic antimicrobials, such as daptomycin^42,44,45^. Upregulation of L-PG production could provide a rationale as to why some of the normal *S. aureus* WT colonies exhibit a rapid increase in MIC against THAM-3ΦG following sub-lethal exposure, but eventually are overwhelmed by the increased levels of THAM-3ΦG. Following exposure to higher levels of THAM-3ΦG, only those cells experiencing the resistance mechanisms associated with the SCV phenotype survived (Fig. 1C).

The SCV phenotype was charsacterized by pin-point size colonies that exhibited a much slower growth rate and MIC up to >16-fold higher for THAM-3ΦG and 4-fold higher for gentamicin (4 mg/L, data not shown) than WT cells. These observations were consistent with *S. aureus* observed following treatment with other antibacterials^46,47^. *S. aureus* SCVs can result from a variety of mutations and exhibit many of the same characteristics as seen in the SCV isolated in this study^30,46–48^. This resistance phenomenon can be attributed in part to the fact that many antibacterials are most effective against cells that are actively dividing, as was seen with THAM-3ΦG^49,50^. Metabolic changes observed in SCV isolates vary^32,33^ and prompted a more in-depth metabolic analysis.

Direct comparison of the metabolic profiles of the WT and SCV (Fig. 6) revealed several metabolic features related to commonly seen SCVs that are auxotrophic for haemin, thymidine, and menadione^31–34^. The high levels of extracellular lactate in the SCV cells grown to mid-log phase (Fig. 7) has been seen in other SCV isolates, including clinical cases, and represents an “early anaerobic” state^33,51^. Some SCVs have been reported to have mutations in the *menB, C, D, E*, or *F* genes coding for enzymes in the menaquinone biosynthesis pathway^35,52–54^. A menaquinone deficiency would impede oxidative phosphorylation and would result in an increased requirement for anaerobic catabolism and energy production. The early production of lactate with a concurrent decrease in cellular pH has been associated with impaired respiratory capacity and the lower cell densities observed in growth profiles of SCV compared to WT^55,56^. This has been observed in *hemB* deficient SCVs, where lactate is the main end product of glucose catabolism - even though the cells are grown in the presence of oxygen under aerobic conditions, a phenomenon referred to as “aerobic glycolysis”^33^. The increased lactate levels that we observed correlated with the reduced acetate and slightly more acidic pH in the SCV media compared to WT media (Fig. 7). Lastly, increased intra- and extracellular levels of uracil suggested a potential thymidine dependence. This is known to happen in *S. aureus* SCV cells with an impaired thymidylate synthase, which is required for the conversion of uracil to thymidine^30,31^.

Of the three potential deficiencies, supplementation assays identified the isolated THAM-3ΦG resistant SCV as a menadione auxotroph. The SCV isolate had almost a full recovery in growth following supplementation with menadione as compared to WT, while supplementation with haemin and thymidine had no effect on cell growth (Fig. 8). Recovery of the cells following menadione supplementation suggests that the SCVs are deficient in the electron transporter, menaquinone^35^. A reduction or loss of menaquinone would result in reduced electron transfer and subsequently lower ATP production and a slower growth rate. The lipid carbon moiety that anchors menaquinone to the lipid bilayer results in menaquinone being very hydrophobic; therefore, menaquinone would have partitioned into the non-polar fraction, which was not initially profiled due to sample complexity and limited ability to identify non-polar metabolites using the Chenomx^TM^ software whose library of small molecule compounds is built for water-soluble metabolites. Subsequent NMR spectral analysis of the intracellular non-polar metabolites showed a lack of NMR signals of menaquinone in the SCV samples (Fig. 8), confirming that the isolated THAM-3ΦG resistant SCV was auxotrophic for menaquinone.

The menaquinone deficiency suggested that a mutation in one or more of the *men* genes had occurred in this *S. aureus* isolate. SCVs auxotrophic for menaquinone have been identified with mutations in the *menB, menC, menD, menE* and *menF* genes coding for enzymes in the menaquinone biosynthesis pathway^35,52–54,57^. Sequencing of the operons associated with these genes, as well as upstream sequences containing putative ribosome binding sites and basal promoter sequences, showed no mutations. However, mutation(s) in a more distal regulatory sequence for one of the operons, or in a gene coding for a protein required for activating operon transcription could be present, as these would not be identified in this work. Insufficient knowledge of the *S. aureus* genome and the regulation of the *men* operons limits our ability to determine which sequences should be examined to identify such a mutation. It should be noted that the remaining enzymes involved in menaquinone synthesis were not investigated, because they are required for the conversion of menadione to menaquinone, and therefore would still have to be functional in order for menadione supplementation to rescue the auxotroph phenotype.

Although THAM-3ΦG is a novel antibacterial selective against *S. aureus*, this study revealed the ability of MRSA to rapidly acquire resistance to THAM-3ΦG through the generation of SCVs auxotrophic for menaquinone and questions the efficacy of its potential clinical use. Previous work showed THAM-3ΦG to be effective in reducing the burden of *S. aureus in vivo*; however, this work also indicated that multiple treatments would be required and potentially at a higher dose^20^. In those studies, colonies were not screened for the SCV phenotype, which may have been missed. Based on the work presented herein, MRSA develops the SCV phenotype very rapidly and a continuous therapy using THAM-3ΦG at high doses *in vivo* may have the same effect. This study highlights the importance of monitoring novel antibacterials for the development of bacterial resistance and the emergence of SCVs, as well as the usefulness of the NMR metabolomics approach to discriminate between different *S. aureus* cellular phenotypes and to identify metabolic changes associated with the development of antimicrobial resistance.

## Materials and Methods

### Bacterial strains

Community-associated MRSA strain LAC, pulsed-field gel-electrophoresis type USA300^12,58,59^, was used in all experiments and is referred to as the wild-type *S. aureus* strain (WT). Experiments were conducted following Biosafety Level 2 guidelines from the CDC Biosafety in Microbiological and Biomedical Laboratories manual, version 5. https://www.cdc.gov/biosafety/publications/bmbl5/

### Synthesis of THAM-3ΦG

The synthesis of THAM-3ΦG described previously^60^ was modified to increase compound yields as follows: 1-BOC hexanediamine (1 equiv.) was initially treated with S-methyl-phenylisourea (1.1 equiv.), prior to attachment to the cyanuric chloride. Following lyophilization, BOC groups were removed using 100% trifluoroacetic acid (TFA) for 2 hours at room temperature. The product (3.6 equiv.) was treated with cyanuric chloride (1 equiv.) in water and refluxed at 120 °C to obtain THAM-3ΦG. THAM-3ΦG was purified by preparative reverse phase HPLC as previously reported (95% acetonitrile, 95% H_2_O, and 0.1% TFA)^60^. THAM-3ΦG stocks were prepared at 5.33 mg/mL in 50% acetonitrile and H_2_O.

### Minimal Inhibitory Concentration (MIC) Testing

The MIC of THAM-3ΦG was determined for each bacterial isolate generated during the resistance assay, and compared to the MIC of the WT *S. aureus* strain, using the microdilution method in 96-well plates with bacteria grown in tryptic soy broth (TSB) according to the guidelines of the British society for Antimicrobial Chemotherapy^61^.

### THAM-3ΦG Resistance Development

The resistance assay was adapted from that of Blair, *et al*.^62^. The *S. aureus* WT strain was cultured to mid-log phase in TSB at 37 °C with shaking at 250 rpm. An aliquot of cells was then inoculated into fresh TSB (diluting 10^2^ to 10^3^-fold) containing a sub-lethal dose of THAM-3ΦG at 1 mg/L. Cells were re-grown to mid-log phase (flask to medium volume ratio of 5:1) and then diluted again into fresh TSB containing a higher dose of THAM-3ΦG. The concentration of THAM-3ΦG was increased by 1 mg/L at each of 11 passages, except for passages 3 and 4 where the concentration of THAM-3ΦG was only increased by 0.25 mg/L. At each passage, samples were frozen, as well as streaked onto LB agar plates, to permit the monitoring of phenotypes and MIC testing at all stages.

### Population Dynamics Assay

Aliquots of frozen cell samples collected during the resistance assay (above) were suspended in 1 mL PBS, diluted as needed, and plated on LB agar. Plates were incubated overnight at 37 °C, and viable cells determined as colony forming units (CFUs) for each phenotype. A dilution containing a minimum of 100 countable CFUs was used to obtain a reliable ratio of normal (WT) to small colony (SC) cells, and the %SC was calculated SC/(SC + WT).

### SCV Stability Assay

The SCV stability assay was modified from that of Singh, *et al*.^63^ as follows: WT cells and SCV cells isolated after treatment with 1 mg/L, 4 mg/L and 64 mg/L THAM-3ΦG in the resistance development assay were each streaked onto TSB agar plates in triplicate and then incubated overnight at 37 °C. A colony was picked and streaked onto fresh TSB agar and re-incubated at 37 °C overnight. This process was repeated for each of the 11 passages. To assess for reversion in the absence of THAM-3ΦG selection, the sizes of the colonies in each passage group were compared to those of WT *S. aureus*. SCV colonies are characteristically one-tenth the size of WT colonies^64^.

### Growth Rates of WT and SCV *S. aureus*

WT and SC *S. aureus* were grown from overnight cultures to an OD_600_ of ~0.45-0.5 in TSB at 37 °C with shaking (250 rpm), and then diluted to ~8.5 × 10^5^ CFU/mL in 50 mL of fresh TSB. Bacterial cell growth was monitored every hour by optical density at 600 nm (OD_600_) over the course of 12 hours.

### Efficacy of THAM-3ΦG against Cells in Stationary Phase of Growth

The activity of THAM-3ΦG against non-growing but metabolically active (stationary phase) WT *S. aureus* cells was evaluated. WT cells grown to mid-log phase were diluted to ~1.5 × 10^6^ CFU/mL into fresh TSB, treated at 8 mg/L THAM-3ΦG (or with an amount of 50% acetonitrile equivalent to that used to dissolve the drug), and then shaken for 5 hours. For nutrient-deprived culture conditions, cells from an overnight culture were removed from the media via centrifugation at 10,000 rpm for 10 min at 4 °C. The “spent” TSB supernatant was then used to dilute cells at a stationary growth stage from an overnight culture to ~1.5 × 10^6^ CFU/mL, which were then treated with 8 mg/L THAM-3ΦG (or an equivalent amount of 50% acetonitrile without THAM-3ΦG) and incubated for 5 hours under the same conditions as the treated mid-log cells. Following the 5-hour incubation, cells were diluted and plated on LB agar. Plates were incubated overnight at 37 °C, and viable cells counted as CFU.

### Metabolic Analysis of WT and SCV *S. aureus* Treated with THAM-3ΦG

WT and SC *S. aureus* cells were grown to mid-log phase in TSB (50 mL in 150 mL flask) at 37 °C with shaking at 250 rpm. Cells were then either immediately harvested by centrifugation (10,000 rpm for 5 min at 4 °C) or diluted into fresh media containing 2 mg/L THAM-3ΦG (or an equivalent amount of acetonitrile) to a final concentration of ~2.3 × 10^7^ CFU/mL. The cultures were further incubated with shaking at 250 rpm for 1 hr at 37 °C, harvested by centrifugation, and resulting cell pellets washed twice with ice-cold PBS. Prior to each harvest, an aliquot of cells was diluted and plated to obtain viable cell counts for normalization of metabolite concentrations. An aliquot (1 mL) of the spent medium supernatant from untreated mid-log cells for both WT and SC were collected following initial centrifugation. Supernatants were passed through 0.2 μm filters and stored at −80 °C. Collected cell pellets were resuspended in 1 mL of 2:1 methanol:chloroform to extract intracellular metabolites following cell lysis^65^. At this and all subsequent steps, samples and reagents were kept on ice.

Cell resuspended in methanol:chloroform were transferred to a 2 mL tube containing 0.7 g of 0.1 mm silica beads. Tubes were placed in an MP Biomedicals FastPrep-24^TM^ 5^G^ and lysed for 2 × 40 sec cycles at a speed of 6.0 m/s, keeping tubes on ice between cycles. Following cell lysis, the resulting slurry was adjusted to a ratio of 1:1:0.2 methanol:chloroform:water by adding 300 μL of chloroform and 300 μL water, and centrifuged for 10 min at 14,000 rpm at 4 °C to separate the aqueous and non-polar phases. The aqueous (top) fraction was removed gently with a glass pipette, and subsequently dried using a vacuum centrifuge overnight with no heat. The organic fraction (bottom phase) was also gently removed and dried down under a stream of N_2_ gas. Dried metabolite mixtures were stored at −80 °C until used for NMR analysis.

For analysis of extracellular metabolites in the growth media, an aliquot (350 μL) of each supernatant from the WT and SC *S. aureus* cultures collected at mid-log phase was treated with 1.4 mL ice-cold acetone, vortexed briefly, and incubated for 1 hour at −20 °C. Resulting samples were centrifuged for 10 min at 14,000 rpm at 4 °C and supernatants transferred to fresh microcentrifuge tubes. Samples were dried by vacuum centrifugation overnight and stored at −80 °C.

### NMR Sample Preparation

For aqueous metabolites, all samples were dissolved in NMR buffer containing 10 mM NaH_2_PO_4_/Na_2_HPO_4_, 0.4 mM imidazole, and 0.25 mM 4,4-dimethyl-4-silapentane-1-sulfonic acid (DSS, chemical shift indicator) in 90% H_2_O/10% D_2_O, pH 7. Intra- and extracellular aqueous metabolites collected from the mid-log growth phase (prior to any treatments) were dissolved in 750 and 650 μL of NMR buffer, respectively. From this metabolite mixture, only 700 and 600 μL were transferred respectively, to each NMR tube to avoid introducing solid debris. Aqueous metabolites from treatment assays (see previous section) were dissolved in 1.1 mL of NMR buffer, spun, and split into two 500 μL aliquots to serve as technical duplicates (of 3 biological replicates). Intracellular non-polar metabolites from all assays were resuspended in 800 µL CDCl_3_ and transferred in their entirety to NMR tubes. Six biological replicate NMR samples were prepared for each treatment condition. All samples were spun at 13,000 rpm for 2 minutes to remove any insoluble debris and then aliquoted into 5 mm NMR tubes (Wilmad Inc.).

### NMR Spectra Collection, Processing, and Metabolite Analysis

One dimensional (1D) ^1^H NMR spectra were recorded at 298 K using a Bruker 600-MHz AVANCE III solution NMR spectrometer equipped with a SampleJet^TM^ automatic sample loading system and a 5 mm liquid-helium-cooled TCI cryoprobe, and the Topspin^TM^ software (Bruker version 3.2) using the Bruker supplied ‘*zgesgp*’ pulse sequence with 256 scans, a ^1^H spectral window of 9600 Hz, 32 K data points, and a dwell time interval of 52 μsec, amounting to an acquisition time of 1.7 sec, and a 1 sec relaxation recovery delay between acquisitions^66^. Since magnetization recovery begins during the acquisition time, the total recovery delay was 2.7 sec. Additional spectra using a total of 4 and 5 sec relaxation recovery delay were also recorded. While the longer delays resulted in a few minor changes in signal intensity, these did not change the metabolite concentrations in WT *S. aureus* relative to the SC variant when comparing signals in spectra measured under the same parameters.

Spectra were processed using Bruker Topspin 3.2 software. Spectral feature analyses and metabolite identifications were undertaken using the Chenomx^TM^ NMR Suite software (version 8.0). Following spectral phasing and baseline corrections, a line broadening function of no more than 0.5 Hz was used, as recommended by published Chenomx protocols and previously reported metabolomics analysis methods^25,67^. The Chenomx shim correction function was applied prior to metabolite analysis. Spectral fitting and metabolite identification and quantification were achieved using the Chenomx^TM^ reference library of small molecule metabolites for 600 MHz magnetic field strength. 4,4-dimethyl-4-silapentane-1-sulfonic acid (DSS, 0.25 mM) was used as an internal standard for metabolite quantification, while imidazole NMR signals were used to correct for small chemical shift differences due to slight pH variations.

### Statistical Analysis

Metabolite concentrations were further normalized to viable cell counts as measured by CFU and volume of NMR buffer. Statistical analysis was performed using the open source software MetaboAnalyst^27,68–71^, whereby concentrations were normalized by log-transformation and auto-scaling (mean centered divided by the standard deviation of each variable) prior to further analysis with MetaboAnalyst statistical analysis modules. Multivariate analysis was performed using principal component analysis (PCA) with established 95% confidence intervals. For hierarchal clustering analysis, distances were measured using a Euclidean correlation and clustering by the Ward algorithm. The most significant 25 metabolites based on t-test or ANOVA were chosen to generate heatmap graphical representations, and were used to represent the key metabolite changes discriminating between WT and SCV phenotypes and the most significant metabolite expression patterns contributing to the separation of WT and SC samples in the 2D-PCA plots.

### Auxotrophism Identification in Small Colony Variant

WT and SCV *S. aureus* cells were grown from overnight cultures to an OD_600_ of ~0.45–0.5 in TSB at 37 °C with shaking (250 rpm), and then diluted to a starting CFU/mL of ~8.5 × 10^5^ in 50 mL of fresh TSB in a 250 mL flask supplemented with either 100 mg/L thymidine, 0.25 mg/L haemin, or 2 mg/L menadione. Cultures were then grown as previously described. Growth was monitored every hour by OD_600_ over the course of 8 hours.

### NMR Analysis of WT and SCV Non-Polar Metabolites Supplemented with Menaquinone

Menaquione-K2 (Sigma-Aldrich) in CDCl_3_ was added to the mid-log non-polar metabolic samples of WT and SCV to a final concentration of 1 mM, and NMR spectra were recollected using the above protocols for collection and processing. An NMR spectrum of a 1 mM menaquinone stock solution was also collected for comparison (Fig. 9).

### DNA Sequencing

Three colony isolates each from SCV selected at 4 mg/L THAM-3ΦG and at 64 mg/L THAM-3ΦG in the resistance development experiment, along with three WT isolates, were grown overnight in TSB. Cells from 1 mL of each culture were pelleted, resuspended in P1 buffer (QIAgen, Germantown, MD), and incubated with 100 µL lysostaphin (0.5 mg/mL). Genomic DNA was extracted using the QIAgen DNeasy Blood and Tissue Kit. PCR amplification of 6 menaquinone biosynthetic pathway genes (menF, menD, menH, menB, menE, and menC) was accomplished using the primers listed in the supplementary material (Table S4). PCR products were both purified and sequenced by GenScript (Piscataway, NJ) using the same primers listed for PCR amplification. Sequencing for each gene was conducted in both forward and reverse directions and included the regions 169 bp upstream of the *menEC* operon and 280 bp upsteam of the *menFDHB* operon coding sequences. Sequences were aligned to the *Staphylococcus aureus* USA300_FPR3757 complete WT genome, NCBI Reference Sequence NC_007793.1^12^ using the Pearson LALIGN program at EMBnet.org.

### Data availability

The datasets generated during and/or analysed during the current study are available from the corresponding author on reasonable request.

## Electronic supplementary material

Supplementary Information
