# Supplementary material for: Exposure of Methicillin-Resistant Staphylococcus aureus to Low Levels of the Antibacterial THAM-3ΦG Generates a Small Colony Drug-Resistant Phenotype
Source: Sci Rep. 2018 Jun 29;8:9850. doi: 10.1038/s41598-018-28283-3 (PMC6026174; doi:10.1038/s41598-018-28283-3)
Supplement: Supplementary file 1 — Supplementary Information [file 41598_2018_28283_MOESM1_ESM.pdf]

**Supplementary Information**

Exposure of Methicillin-Resistant *Staphylococcus aureus* to Low Levels of the Antibacterial THAM-3ΦG Generates a Small Colony Drug-Resistant Phenotype

Alan J. Weaver Jr.[1*], Tami R. Peters,[1] Brian Tripet,[1] Abigail Van Vuren[1], Rakesh[2], Richard E. Lee[2], Valérie Copié[1], Martin Teintze[1]

## SUPPORTING INFORMATION

**Table S1. Passage number, Passage Concentration of THAM-3ΦG, and Minimum Inhibitory Concentrations of Cell Isolates.**

| Passage No. | Passage [T3PG] | MIC (mg/L) | n |
|---|---|---|---|
| P-0 | 0 mg/L | 2 | 48 |
| P-1L | 1 mg/L | 4 | 8 |
| P-1S | | 8 | 10 |
| P-2L | 2 mg/L | 4 | 6 |
| P-2S | | 8 | 12 |
| P-3L | 2.5 mg/L | 4 | 6 |
| P-3S | | 8 | 12 |
| P-4L | 3 mg/L | 4 | 6 |
| P-4S | | 8 | 8 |
| P-5L | 4 mg/L | 4 | 6 |
| P-5S | | 8 | 6 |
| P6 | 5 mg/L | 8 | 6 |
| P7 | 6 mg/L | 8 | 9 |
| P8 | 8 mg/L | 32 | 11 |
| P9 | 16 mg/L | 32 | 8 |
| P10 | 32 mg/L | 32 | 9 |
| P11 | 64 mg/L | 64 | 7 |

With each passage, the concentration of THAM-3ΦG was increased, which is reflected as the passage concentration. MICs were recorded for each cell type (denoted by L or S for large and small) isolated in the resistance assay. MIC is greater than or equal to the concentration of THAM-3ΦG at each passage. The number of technical replicates for each MIC test is represented by n. A minimum of 3 biological replicates were conducted for each isolate.

**Table S2. Loading Factors from the PCA of WT and SCV, Treated and Untreated with THAM-3ΦG**

| Metabolite | PC1 | PC2 |
|---|---|---|
| 2-Hydroxyisobutyrate | -0.21931 | 0.066952 |
| AMP | 0.20476 | 0.10556 |
| Acetate | -0.09675 | 0.14288 |
| Alanine | -0.21148 | 0.041895 |
| Asparagine | -0.04029 | 0.30841 |
| Aspartate | 0.19171 | 0.19284 |
| Betaine | 0.078021 | 0.30806 |
| Choline | -0.04716 | 0.16751 |
| Cystathionine | -0.05587 | 0.082503 |
| Dimethylamine | -0.15678 | 0.18598 |
| Formate | -0.21685 | 0.10983 |
| Glucose-1-phosphate | -0.24133 | -0.0313 |
| Glutamate | 0.085426 | 0.27294 |
| Glutamine | -0.12531 | 0.048285 |
| Glycine | -0.20279 | 0.10716 |
| Isoleucine | -0.16718 | 0.23029 |
| Isovalerate | -0.19481 | 0.003794 |
| Lactate | 0.16269 | 0.23403 |
| Leucine | -0.18304 | 0.20341 |
| Lysine | -0.24527 | -0.01398 |
| Methionine | -0.07726 | 0.23719 |
| NAD | 0.20178 | 0.16067 |
| NADP | 0.20042 | 0.18237 |
| Niacinamide | 0.15796 | 0.11186 |
| Phenylalanine | -0.22942 | 0.10733 |
| Proline | 0.18245 | 0.16791 |
| Succinate | -0.23823 | 0.015148 |
| Trimethylamine | 0.16618 | 0.23992 |
| Trimethylamine N-oxide | 0.11707 | 0.28054 |
| UDP-galactose | -0.24 | 0.023353 |
| Valine | -0.16669 | 0.21628 |
| sn-Glycero-3-phosphocholine | -0.13242 | 0.25066 |
| pi-Methylhistidine | -0.16544 | 0.024453 |

**Table S3. Loading Factors from the PCA of WT and SCV at Mid-log Phase.**

| Metabolite | PC1 | PC2 |
|---|---|---|
| 2-Hydroxyisobutyrate | -0.21931 | 0.066952 |
| AMP | 0.20476 | 0.10556 |
| Acetate | -0.09675 | 0.14288 |
| Alanine | -0.21148 | 0.041895 |
| Asparagine | -0.04029 | 0.30841 |
| Aspartate | 0.19171 | 0.19284 |
| Betaine | 0.078021 | 0.30806 |
| Choline | -0.04716 | 0.16751 |
| Cystathionine | -0.05587 | 0.082503 |
| Dimethylamine | -0.15678 | 0.18598 |
| Formate | -0.21685 | 0.10983 |
| Glucose-1-phosphate | -0.24133 | -0.0313 |
| Glutamate | 0.085426 | 0.27294 |
| Glutamine | -0.12531 | 0.048285 |
| Glycine | -0.20279 | 0.10716 |
| Isoleucine | -0.16718 | 0.23029 |
| Isovalerate | -0.19481 | 0.003794 |
| Lactate | 0.16269 | 0.23403 |
| Leucine | -0.18304 | 0.20341 |
| Lysine | -0.24527 | -0.01398 |
| Methionine | -0.07726 | 0.23719 |
| NAD | 0.20178 | 0.16067 |
| NADP | 0.20042 | 0.18237 |
| Niacinamide | 0.15796 | 0.11186 |
| Phenylalanine | -0.22942 | 0.10733 |
| Proline | 0.18245 | 0.16791 |
| Succinate | -0.23823 | 0.015148 |
| Trimethylamine | 0.16618 | 0.23992 |
| Trimethylamine N-oxide | 0.11707 | 0.28054 |
| UDP-galactose | -0.24000 | 0.023353 |
| Valine | -0.16669 | 0.21628 |
| sn-Glycero-3-phosphocholine | -0.13242 | 0.25066 |
| pi-Methylhistidine | -0.16544 | 0.024453 |

**Table S4. Primers Used for PCR Amplification and Gene Sequencing.**

| Primer Name | Gene Target | Primer sequence: 5' - 3' |
|---|---|---|
| TP-menFF | menF | CGAAGAAAGAAGTGAAATGGATGGCTACGGGCG |
| TP-menFR | menF | GCTTCAAATGCAAGTGCAAGTGGCGTTGAGCG |
| TP-menDF | menD | GTACGGCGTAAGGGAAGTAGTTATCAGTCCG |
| TP-menDR | menD | CGGTCTAACAACGTCGTAATATAATCAAAATTCCACG |
| TP-menHF | menH | GCCACATTGTTATCTGAAACTTCGACG |
| TP-menHR | menH | GCGAGGGATTTGGTCTTCACCTACATAACC |
| TP-menBF | menB | GGGAAAAGTTGCCATTATTTCAATCGC |
| TP-menBR | menB | GGTTGGCGAGAATTCAATTTCTCACCAGCTCG |
| TP-menEF | menE | CATAACCACTGCTAGAATGTCATAAAATCG |
| TP-menER | menE | CGGTGTTACAATTTGTGACTTAAATGGCTC |
| TP-menCF | menC | GCGAAGCAATTTCCAGGTATCAGTGACGC |
| TP-menCR | menC | GGCGGCTTTGACCCTGTTCCG |
